# A novel short-term outcome prediction model for esophagectomy patients receiving neoadjuvant immunochemotherapy and neoadjuvant chemotherapy: a muti-center retrospective study

**DOI:** 10.3389/fonc.2026.1805033

**Published:** 2026-05-29

**Authors:** Qi-Hong Zhong, Ye-Qin Zhang, Jing-Yu Wu, Jiang-Shan Huang, Fei-Long Guo, Wen-Wei Lin, Sui Chen, Gang Wang, Zhen-Yang Zhang, Jiang-Bo Lin, Mao-Xiu Yuan

**Affiliations:** 1 Department of Thoracic Surgery, Fujian Medical University Union Hospital, Fuzhou, Fujian, China; 2 Department of Thoracic Surgery, Ganzhou Cancer Hospital, Ganzhou, Jiangxi, China; 3 Department of Thoracic Surgery, Affiliated Hospital of Jinggangshan University, Ji’an, Jiangxi, China

**Keywords:** esophageal cancer, esophagectomy, multicenter, neoadjuvant immunochemotherapy, short-term outcome

## Abstract

**Background:**

Due to the lack of a comprehensive evaluation of the short-term prognosis of patients undergoing radical resection of esophageal cancer after neoadjuvant therapy, recent clinical strategies have remained subjective and controversial. The recognition of pretreatment risk factors and tailored treatment could improve outcomes of esophagectomy patients. Therefore, we aimed to develop a predictive model that differentiates high-risk conditions after surgery in patients with esophageal cancer receiving neoadjuvant therapy.

**Methods:**

We conducted a muti-center, retrospective cohort study in Fujian Medical University Union Hospital, Ganzhou Cancer Hospital and Affiliated Hospital of Jinggangshan University. A principal component (PC) analysis was applied for data simplification and the extraction of patient short-term outcome characteristic. We identified risk status on admission and operation, via a logistic regression and then constructed prediction models for worsened short-term outcomes.

**Results:**

Of 334 patients of train set underwent neoadjuvant therapy and esophagectomy, 142(42.5%) received neoadjuvant chemotherapy (NC), 192(57.5%) received neoadjuvant immunochemotherapy (NIC). After grouping by principal component analysis, patients were divided into high-risk group (83,25%) and low-risk group (251,75%). Twelve features regarding clinical feature, nutrition indicators, laboratory indicators and intraoperative data were identified. The prediction model showed the best performance in predicting high short-term outcome risk, with an area under the receiver operating characteristic curve (AUC) of 0.794 (95%CI, 0.712,0.876) in NC, and 0.781 (95%CI, 0.705,0.858) in NIC.

**Conclusion:**

A novel short-term outcome prediction model, offers a comprehensive assessment of posttreatment recovery in esophagectomy patients after neoadjuvant immunotherapy combined with chemotherapy and neoadjuvant chemotherapy.

## Background

Esophageal squamous cell carcinoma is among the most invasive malignant tumors and ranks as the sixth leading cause of cancer-related deaths worldwide (1). Post neoadjuvant esophagectomy is one of the principal treatment modalities for locally advanced esophageal cancer (2). However, due to the complexity of esophagectomy within the spectrum of gastrointestinal surgeries, patients are at an elevated risk for postoperative complications and mortality. Therefore, precise preoperative risk assessment is paramount for ameliorating short-term outcomes. With the recent advent of immunotherapy, the combination of neoadjuvant chemotherapy and immunotherapy for esophageal cancer has garnered increased attention, and a multitude of studies have indicated that preoperative neoadjuvant chemotherapy coupled with immunotherapy can enhance pathological response rates and mitigate the risk of postoperative recurrence (3–5).

Perioperative management significantly influences postoperative complications and enhances recovery after surgery (ERAS) in esophageal cancer patients. Research has revealed that meticulous perioperative management can augment preoperative immune function and overall physical defense, bolster surgical tolerance, and expedite patient recovery. This can lead to reduced hospitalization periods, diminished healthcare expenditures, and a lower incidence of postoperative complications (6, 7).

Principal component analysis (PCA) is a sophisticated statistical technique commonly applied for dimensionality reduction, linear correlation elucidation and data simplification (8–10). Prior studies have predominantly focused on the relationship between preoperative status and a single complication (11–14). However, a comprehensive scoring system tailored to assess the integrated short-term prognosis of esophageal cancer patients after neoadjuvant therapy has yet to be developed. Identifying patients at high risk for complications and intervening preoperatively with precision is a critical issue that warrants immediate attention.

In this study, we utilized stratified analysis to elucidate the risk factors that impact short-term prognosis following esophagectomy in patients who underwent neoadjuvant chemotherapy with or without immunotherapy. Moreover, we developed a predictive model to distinguish high-risk scenarios following esophagectomy for patients treated with neoadjuvant chemotherapy and immunotherapy, aiming to inform and refine clinical management strategies.

## Methods

### Patient population

**Figure 1:**
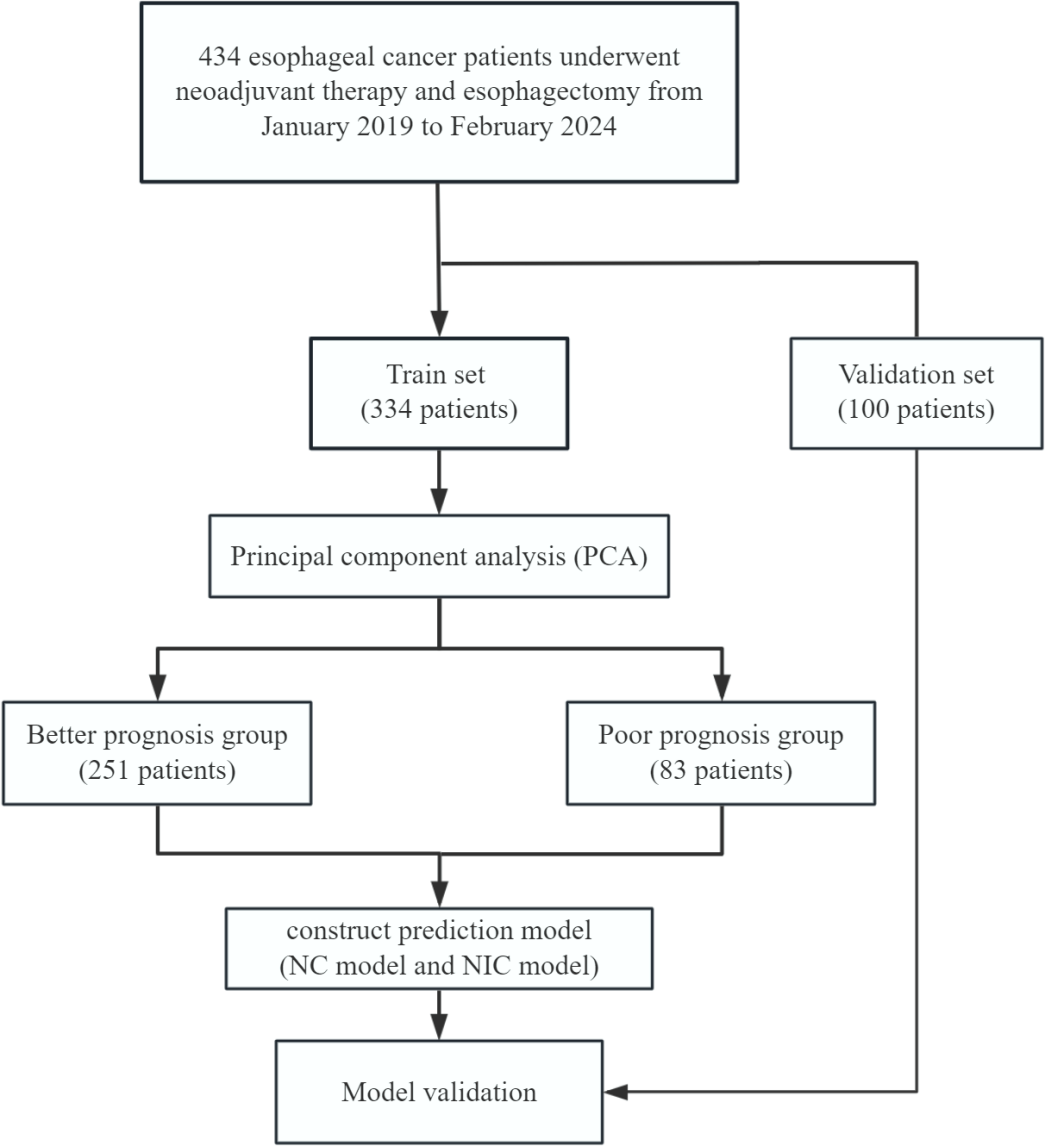
Work flow. NC, neoadjuvant chemotherapy; NIC, neoadjuvant immunochemotherapy.

A total of 434 patients who were diagnosed with esophageal cancer and who underwent neoadjuvant therapy and esophagectomy at Fujian Medical University Union Hospital, Ganzhou Cancer Hospital and Affiliated Hospital of Jinggangshan University from January 2019 to February 2024 were included in this retrospective study. Patients from Fujian Medical University Union Hospital were included in the training set (n=334), and patients from Ganzhou Cancer Hospital and Affiliated Hospital of Jinggangshan University were used as the validation set (n=100) (**Figure 1**).

The inclusion criteria: (1) esophageal squamous cell carcinoma was confirmed by preoperative gastroscopy; (2) no distant metastasis was found in preoperative examination and postoperative pathology; (3) patients receiving two or more cycles of neoadjuvant PD-1 inhibitor and chemotherapy entered the neoadjuvant immunochemotherapy group, patients receiving neoadjuvant chemotherapy alone entered the chemotherapy group. Exclusion criteria: (1) previous history of cancer or malignant tumors in other parts of the body; (2) patients with severe lung diseases; (3) esophagectomy with mediastinoscopy; (4) thoracotomy or laparotomy for esophageal cancer resection; (5) Incomplete data. All of the patients provided written informed consent for the procedure. All of the patients provided written informed consent for the procedure, and the study protocol was approved by the Institutional Review Board of Fujian Medical University Union Hospital (Approval No. 2024KY037) and Affiliated Hospital of Jinggangshan University (Approval No. 2024115).

### Clinical characteristics and laboratory tests

The baseline demographic and clinical characteristics included sex, age, body mass index (BMI), register, American Society of Anesthesiologists (ASA) score, tumor location, and history of alcohol consumption and smoking. Laboratory test results, including the white blood cell (WBC) count, carcinoembryonic antigen (CEA) level, hemoglobin (Hb) level, platelet (PLT) count, neutrophil, lymphocyte, monocyte, albumin, total cholesterol, triglyceride and neutrophil-to-lymphocyte ratio (NLR), were recorded in our database for esophageal cancer patients. Categorical variables, especially total cholesterol, triglyceride and neutrophil, were sorted by 75% quartile.

### Nutrition indicator

Nutritional indicators, including the prognostic nutritional index (PNI), the geriatric nutritional index (GNRI) and the systemic immune-inflammatory index (SII), were calculated as follows: PNI = 10 × serum albumin (g/dL) + 0.005 × total lymphocyte count (per mm^3^) (13), GNRI = 1.489 × albumin (g/dl) + 41.7 × usual weight/ideal weight (14), and SII = platelet × neutrophil/lymphocyte counts (15).

### Intraoperative factors and short-term outcome data

Intraoperative data, including blood loss, operation time, number of lymph nodes dissected and number of positive lymph nodes, were collected from surgical records. Postoperative pathology data were obtained from postoperative pathological records following the eighth edition of the TNM staging system (16). Postoperative short-term outcome data, including pulmonary infection, anastomotic fistula, blood transfusion, intensive care unit (ICU) management, anastomotic leakage, length of stay (LOS), duration of chest tube drainage and duration of neck drainage tube placement, were collected from the patients’ medical records and course notes. All postoperative complications were diagnosed by experienced physicians. The severity of complications was graded according to the Clavien–Dindo (CD) system (17).

### Principal component analysis

Principal component analysis (PCA) was performed with R software (4.3.3), which simplified the analysis by expressing short-term outcome indicators with six dimensions (**Supplementary Figure 1**). We chose dimension 1, which had the highest percentage of variance, as the parameter for explaining prognosis. The following novel PC score system was used: PC score = 0.450 × LOS + 0.434 × duration of chest tube drainage + 0.406 × duration of neck drainage tube + 0.419 × ICU stay time + 0.426 × CD score + 0.296 × anastomotic leakage. We further categorized patients according to quartile PC score as follows: a better prognosis group (below the 75% quartile) and a poor prognosis group (above the 75% quartile).

### Statistical analysis

Differences between the groups were compared using the chi-square test or Fisher’s exact test for categorical variables. For normally distributed continuous variables, we used an independent t test. For continuous nonparametric variables, the Mann-Whitney U test was applied to analyze the differences. Receiver operating characteristic (ROC) curves and the area under the curve (AUC) were calculated to assess the accuracy of the predictive models. Statistical analyzes were performed with SPSS software (version 23.0, SPSS, Inc.) and R software (version 4.3.3).

## Results

### Baseline information

Of the 334 patients in the training set diagnosed with esophageal cancer who underwent neoadjuvant therapy and esophagectomy, 142 (42.5%) received neoadjuvant chemotherapy (NC), while 192 (57.5%) received neoadjuvant immunochemotherapy (NIC). Of the 100 patients in the validation set, 50 (50%) received NC, and 50 (50%) received NIC. No marked differences were observed between the groups for the baseline parameters, including age, sex, BMI, registry, smoking status, history of alcohol consumption and comorbidity status (all p values > 0.05, **Table 1**).

**Table 1:** Compared different baseline information between subgroups.

Variables	Subgroups of train set			Train set (n=334)	Validation set (n=100)	p value
	NC therapy (n=142)	NIC therapy (n=192)	p value			
Age, mean (SD)	61.32(7.26)	60.46(6.71)	0.457	60.83(6.95)	60.18(6.43)	0.132
Gender (male), n (%)	108(76.1%*)	154(80.2%*)	0.362	262(78.4%**)	82(82.0%**)	0.442
BMI	22.22(2.71)	21.51(2.79)	0.874	21.81(2.77)	21.98(3.07)	0.144
Register(urban), n (%)	115(81.0%*)	167(87.0%*)	0.135	282(84.4%**)	85(15.0%**)	0.890
Smoke (+), n (%)	76(53.5%*)	115(59.9%*)	0.224	191(57.2%**)	60(60.0%**)	0.617
Drink (+), n (%)	43(30.3%*)	61(31.8%*)	0.771	104(31.1%**)	31(31.0%**)	0.979
Comorbidity (+), n (%)	39(27.7%*)	52(27.4%*)	0.953	91(27.2%**)	28(28.0**)	0.882

The “*” refers to the proportion in the training set(n=334), and “**” refers to the proportion in the overall population(n=442), BMI: body mass index.

### PCA and prognostic distinction

Principal component analysis (8) (PCA) transforms the ICU stay time, CD score, anastomotic leakage, length of stay (LOS), duration of chest tube drainage and duration of neck drainage into a set of principal components through linear transformation. After PCA dimension reduction, six dimensions were derived from these short-term outcome variables. Furthermore, dimension 1, which had the highest percentage of variance (58.6%), was selected as the parameter for calculating the PC score (**Supplementary Table 1**). According to the 75% quartile PC score (PC score=17.6783), we divided the patients in the training set into a better prognosis group (n=251) and a poor prognosis group (n=83). In the validation set, we grouped the samples in the same way and with the same cut-off points. For patients in the training set and validation set, the incidence of postoperative complications in the better prognosis group was significantly lower, and these patients also recovered significantly faster (all p values <0.05, **Table 2**). This finding demonstrated that the PC score makes a clear distinction between the short-term prognosis of patients.

**Table 2:** Compared different short-term outcomes between subgroups of Train set and Validation set.

Variables	Train set (n=334)				Validation set (n=100)		
	Better prognosis (n=251)	Poor prognosis (n=83)	p value		Better prognosis (n=59)	Poor prognosis (n=41)	p value
Pulmonary infection (+), n (%)	60(23.9%)	46(55.4%)	**<0.001**	13(22.0%)	20(48.8%)	**0.005**
Anastomotic fistula (+), n (%)	16(6.4%)	33(39.8%)	**<0.001**	3(5.1%)	10(24.4%)	0.007**
Blood transfusion (+), n (%)	5(2.0%)	8(9.6%)	**0.002**	1(1.7%)	6(15.0%)	0.016**
ICU management (+), n (%)	47(18.7%)	54(65.1%)	**<0.001**	35(59.3%)	33(80.5%)	**0.026**
Anastomotic leak (+), n (%)	16(6.4%)	33(39.8%)	**<0.001**	4(6.8)	10(24.4%)	0.014**
Length of stay, Medium (IQR)	9(2)	23(16)	<0.001*	15(9)	25(15)	<0.001*
Duration of chest tube drainage, Medium (IQR)	8(2)	14(7)	<0.001*	9(3)	14(5)	<0.001*
Duration of neck drainage tube, Medium (IQR)	6(3)	11(7)	<0.001*	8(4)	12(6)	<0.001*
ICU stay time, Medium (IQR)	0(0)	5(9)	<0.001*	4(5)	6(7)	<0.001*
CD score, (>2), n (%)	20(8.0%)	55(66.3%)	**<0.001**	6(10.2%)	27(65.9%)	**<0.001**

Values marked with “*” were compared using Kruskal-Wallis rank-sum test.

Values marked with “**” were adjusted p-values

IQR, InterQuartile Range.

ICU, Intensive Care Unit; CD, Clavien-Dindo.

The bold values represent the p values < 0.05.

### Univariate and multivariate analyzes of risk factors

Univariate analysis of baseline information, clinical features, laboratory indicators, intraoperative data and postoperative pathology data revealed that in NC patients, poor prognosis was closely related to WBC (p=0.002), increased neutrophil (p=0.007), albumin (p=0.001), and NLR (p=0.038) counts and increased operation time (p=0.007) (**Table 3**). In NIC patients, a poor prognosis was closely related to an urban registry (p=0.011), the presence of weight loss (p=0.024), an ASA score =3 (p=0.021), a lymphocyte count (p=0.046), a higher total cholesterol level (p=0.008), a higher triglyceride level (p=0.042) and a greater number of lymph nodes dissected (p=0.002) (**Table 3**).

**Table 3:** univariate analysis of baseline information clinical features, laboratory indicators, intraoperative data and postoperative pathology data.

Variables	Neoadjuvant chemotherapy (n=142)				Neoadjuvant immunochemotherapy(n=192)		
	Better prognosis (n=106)	Poor prognosis (n=36)	p value		Better prognosis (n=145)	Poor prognosis (n=47)	p value
Baseline information							
Sex (male), n (%)	82(77.4%)	26(72.2%)	0.533	112(77.2%)	42(89.4%)	0.070
Age (>60), n (%)	56(52.8%)	20(55.6%)	0.777	67(46.2%)	24(51.1%)	0.562
Register(urban), n (%)	84(79.2%)	31(86.1%)	0.364	121(83.4%)	46(97.9%)	0.011**
Smoke (+), n (%)	56(52.8%)	20(55.6%)	0.777	85(58.6%)	30(63.8%)	0.527
History of drinking (+), n (%)	34(32.1%)	9(25.0%)	0.425	43(29.7%)	18(38.3%)	0.269
Lose weight (+), n (%)	12(11.3%)	6(16.7%)	0.405	13(9.0%)	10(21.3%)	**0.024**
BMI, mean (SD)	22.28(2.90)	22.02(2.08)	0.621	21.57(2.91)	21.34(2.39)	0.303
Clinical features							
ASA score, n (%)			0.999**			0.021**
1	3(2.8%)	1(2.8%)		2(1.4%)	2(4.3%)	
2	85(80.2%)	29(80.6%)		133(91.7%)	36(76.6%)	
3	18(17.0)	6(16.7%)		10(6.9%)	9(19.1%)	
Tumor location, n (%)			0.139**			0.242**
Upper thoracic	10(9.4%)	2(5.6%)		14(9.7%)	3(6.4%)	
Middle thoracic	57(53.8%)	14(38.9%)		66(45.5%)	28(59.6%)	
Lower thoracic	39(36.8%)	20(55.6%)		65(44.8%)	16(34.0%)	
Laboratory indicators							
CEA (ng/ml), mean (SD)	2.95(3.88)	2.99(1.52)	0.929	2.58(1.54)	2.39(1.00)	0.419
Hb (g/L), mean (SD)	126.71(12.85)	123.39(16.47)	0.276	123.74(18.73)	122.66(19.13)	0.733
PLT (10^9^/L), mean (SD)	221.29(60.55)	217.94(48.35)	0.738	214.96(61.82)	213.19(66.42)	0.867
WBC (10^9^/L), mean (SD)	5.97(1.63)	5.13(1.23)	**0.002**	6.08(1.94)	5.70(1.85%)	0.237
Neutrophil (>75%quartile), n (%)	32(30.2%)	3(8.3%)	0.007**	3.73(1.62)	3.54(1.59)	0.481
Lymphocyte, mean (SD)	1.76(0.54)	1.72(0.63)	0.763	1.71(0.55)	1.55(0.46)	**0.046**
Monocyte, mean (SD)	0.44(0.17)	0.39(0.12)	0.105	0.43(0.20)	0.44(0.18)	0.829
Albumin (g/L), mean (SD)	42.18(3.87)	39.31(3.16)	**0.001**	41.86(3.90)	41.46(3.82)	0.532
Total cholesterol, (>75% quartile), n (%)	24(22.6%)	8(22.9%)	0.979	39(26.9%)	4(8.5%)	0.008**
Triglyceride, (>75% quartile), n (%)	38(36.2%)	10(28.6)	0.411	31(21.4%)	17(36.2%)	**0.042**
NLR, mean (SD)	2.28(1.48)	1.88(0.75)	**0.038**	2.48(2.16)	2.43(1.17)	0.841
Intraoperative data							
Blood loss (ml) Medium (IQR)	100(50)	100(50)	0.145*	100(50)	100(50)	0.866*
Operation time, (> Medium), n (%)	60(56.6%)	11(30.6%)	**0.007**	327(74)	326(84)	0.937*
Lymph nodes dissected number, Medium (IQR)	31.5(17)	34(13)	0.354	36(18)	26(18)	0.002*
Positive lymph nodes number, Medium (IQR)	1(3)	0(2)	0.612	1(2)	1(3)	0.273*
Postoperative pathology							
T stage			0.752**			0.598
0	7(6.6%)	3(8.3%)		40(27.6%)	11(23.4%)	
1	16(15.1%)	6(16.7%)		31(21.4%)	7(14/9)	
2	27(25.5)	6(16.7)		20(13.8%)	9(19.1%)	
3	56(52.8%)	21(58.3%)		54(37.2%)	20(42.6%)	
N stage			0.350**			0.317**
0	51(48.1%)	18(50%)		75(51.7%)	21(44.7%)	
1	27(25.5%)	12(33.3%)		46(31.7%)	13(27.7%)	
2	22(20.8%)	3(8.3%)		20(13.8%)	12(25.5%)	
3	6(5.7%)	3(8.3%)		4(2.8%)	1(2.1%)	
M stage (0), n (%)	106(100%)	36(100%)	1.000	145(100%)	47(100%)	1.000

Values marked with “*” were compared using Kruskal-Wallis rank-sum test.

Values marked with “**” were adjusted p-values.

The bold values represent the p values < 0.05.

IQR, InterQuartile Range; ASA, American Society of Anesthesiologists; NLR, Neutrophils/lymphocytes; WBC, White blood cells; Hb, hemoglobin; CEA, carcinoembryonic antigen; PLT, blood platelet.

Through multivariate logistic regression, independent risk factors for NC patients, including the serum ALB concentration (OR = 0.829) and a longer operation time (OR = 0.317), were identified (p<0.05, **Table 4**). For NIC patients, an urban registry (OR = 8.487), an ASA score =3 (OR = 3.337), a high total cholesterol level (OR = 0.029), a high triglyceride level (OR = 2.61), a high lymphocyte count (OR = 2.255) and a large number of lymph nodes dissected (OR = 1.032) were identified as independent risk factors for poor prognosis (p<0.05, **Table 4**).

**Table 4:** Multivariate regression of Neoadjuvant chemotherapy and Neoadjuvant immunochemotherapy patients.

Variables	Neoadjuvant chemotherapy		Neoadjuvant immunochemotherapy	
	OR (95% CI)	p value	OR (95% CI)	p value
Register, (urban)			8.487[1.058,68.078]	**0.044**
ASA score, (3)			3.337[1.122,9.923]	**0.030**
Lose weight (+)			1.997[0.711,5.610]	0.189
Albumin, g/L	0.829 [0.739,0.929]	**0.001**		
Total cholesterol, (>6.5mmol/L)			0.279[0.088,0.880]	**0.029**
Triglyceride, (>75% quartile)			2.610[1.157,5.885]	**0.021**
WBC, (10^9^/L)	0.816[0.545,1.223]	0.324		
Neutrophil, (>75% quartile)	0.552[0.099,3.078]	0.498		
NLR	0.766[0.411,1.330]	0.343		
Lymphocyte, (10^9^/L)			0.444[0.202,0.974]	**0.043**
Lymph nodes dissected number			0.969[0.943,0.995]	**0.018**
Operation time, (> medium)	0.371[0.155,0.887]	**0.026**		

ASA, American Society of Anesthesiologists; WBC, White blood cells; Hb, hemoglobin; NLR, Neutrophils/lymphocytes.

The bold values represent the p values < 0.05.

### Novel prediction model construction, comparison with previous nutrition indicators and model validation

**Figure 2:**
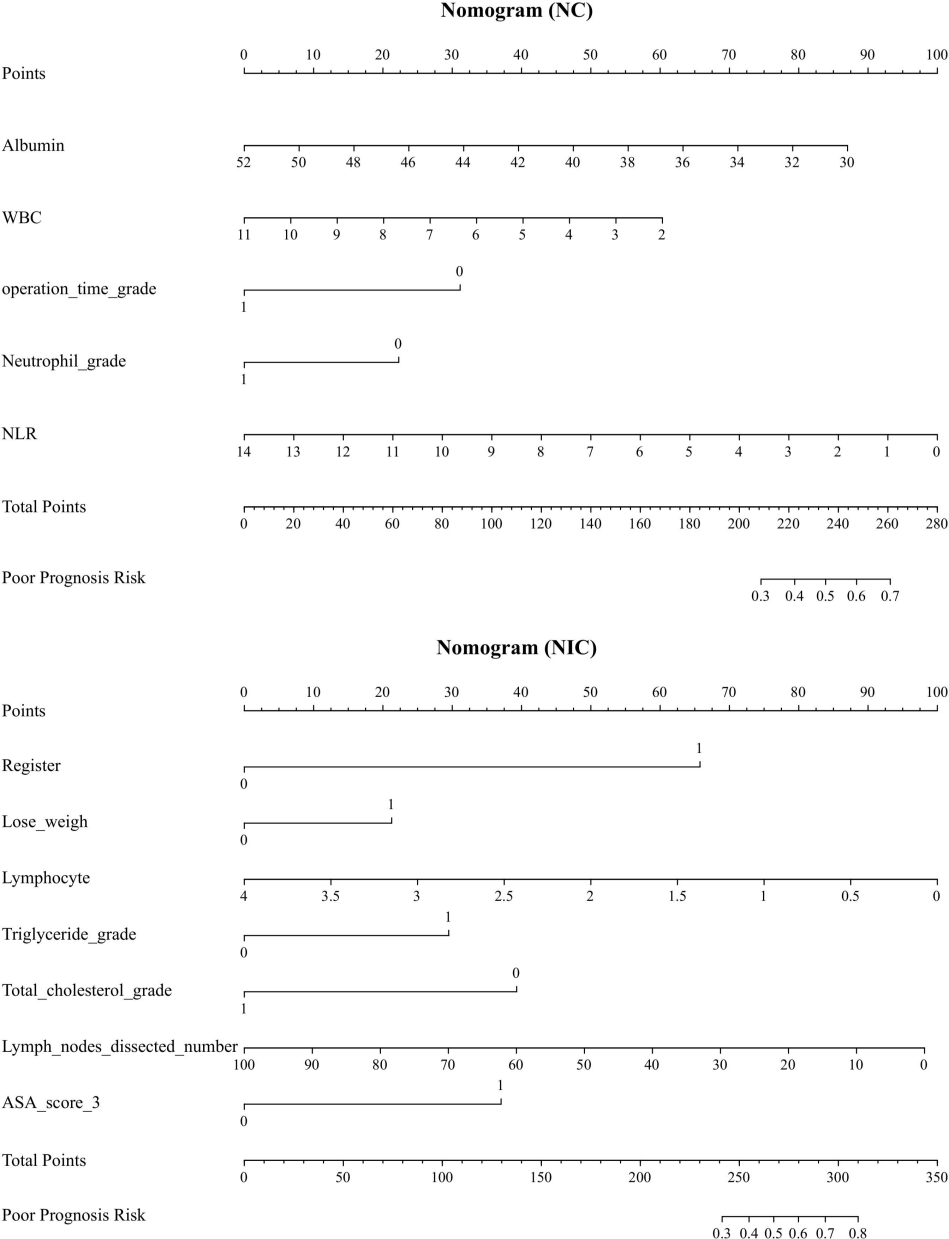
Nomogram of NC and NIC prediction models. ASA, American Society of Anesthesiologists; WBC, White blood cells; Hb, hemoglobin; NLR, Neutrophils/lymphocytes; NC, neoadjuvant chemotherapy; NIC, neoadjuvant immunochemotherapy.

Based on the regression coefficients obtained for each factor in multivariate logistic regression, we constructed a novel management model for NC and NIC patients: risk score _NC_= – 0.188*Albumin– 0.203*WBC– 0.267 * NLR– 0.595 * Neutrophil– 0.993 * Operation time, and risk score _NIC_= 2.138 * register + 1.205 * ASA + 0.692 * lose weight + 0.959 * Triglyceride– 1.278 * Total cholesterol– 0.813 * Lymphocyte– 0.032 * Lymph nodes dissected number. A nomogram was also drawn to accurately calculate the probability of poor prognosis (**Figure 2**). In addition, we constructed a checklist to promote the application of the model (**Table 5**).

**Table 5:** Checklist of prediction model.

Variables	Parameter	Condition
Neoadjuvant chemotherapy		
Albumin	–0.188	Continuous variables
WBC	–0.203	Continuous variables
NLR	–0.267	Continuous variables
Neutrophil	–0.595	>4.173/L
Operation time	–0.993	>328.5 minutes
Neoadjuvant immunochemotherapy		
Register	2.138	Urban
ASA score	1.205	Score=3
Lose weight	0.692	Positive
Triglyceride	0.959	>1.675mmol/L
Total cholesterol	–1.278	>6.415mmol/L
Lymphocyte	–0.813	Continuous variables
Lymph nodes dissected number	–0.032	Continuous variables

WBC, White Blood Cell; NLR, Neutrophil to lymphocyte ratio; ASA, American society of Anesthesiologists.

**Figure 3:**
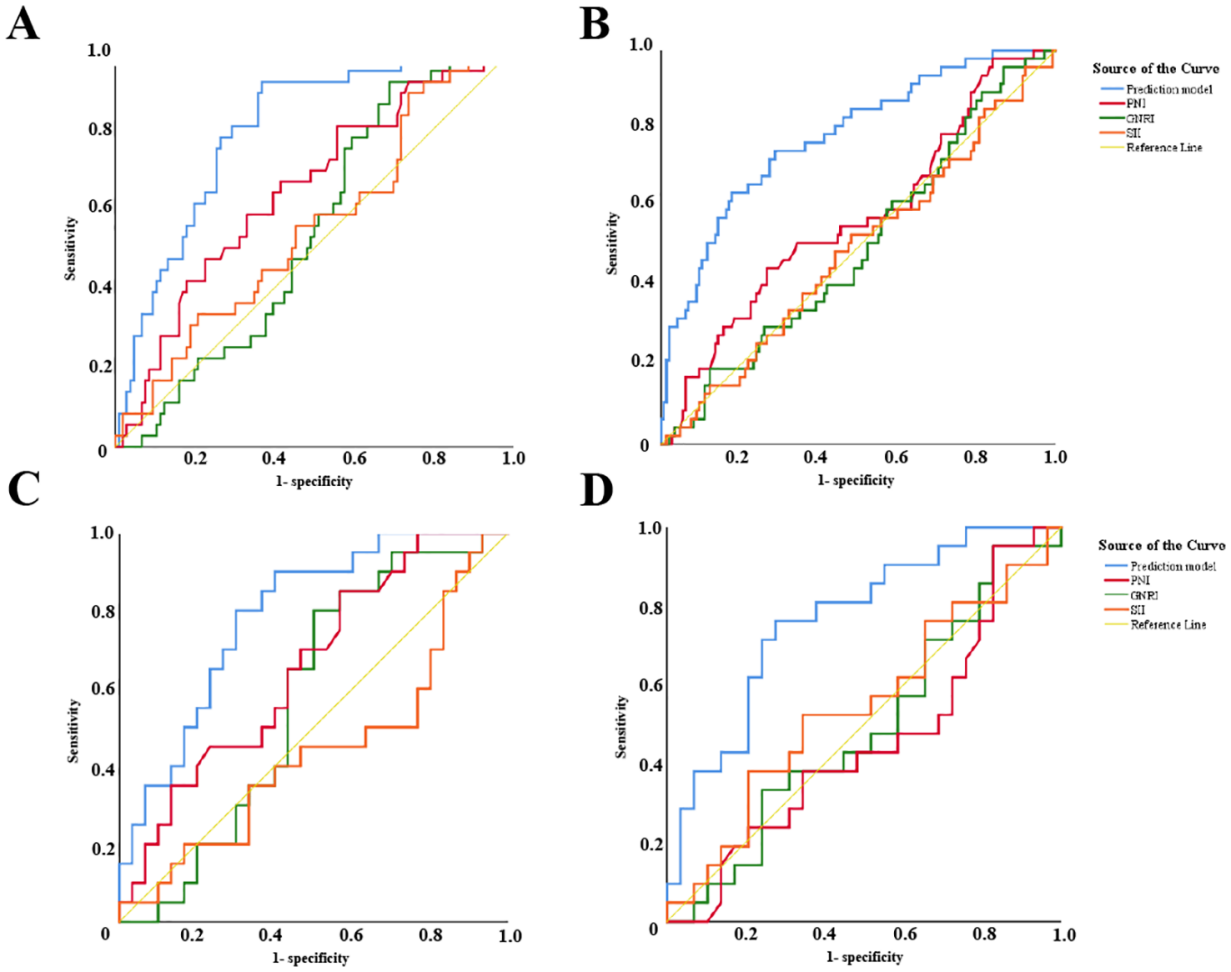
ROC curves of prediction model, GNRI, PNI and SII in train set and validation set. **(A)** Receiver operating characteristic curves of NC patients; The AUC of prediction model was 0.794(95% CI, 0.712,0.876); the AUC of GNRI was 0.539 (95% CI, 0.411,0.638); the AUC of SII was 0.553 (95% CI, 0.445,0.661); the AUC of PNI was 0.645(95% CI, 0.541,0.749). **(B)** Receiver operating characteristic curves of NIC patients; The AUC of prediction model was 0.781(95% CI, (0.705,0.858); the AUC of GNRI was 0.502 (95% CI, 0.409,0.595); the AUC of SII was 0.492(95% CI, 0.397,0.588); the AUC of PNI was 0.574 (95% CI, 0.479,0.670). **(C)** Receiver operating characteristic curves of NC patients; The AUC of prediction model was 0.788 (95% CI, 0.664,0.913); the AUC of GNRI was 0.573 (95% CI, 0.414,0.733); the AUC of SII was 0.438 (95% CI, 0.271,0.606); the AUC of PNI was 0.654(95% CI, 0.502,0.806). **(D)** Receiver operating characteristic curves of NIC patients; The AUC of prediction model was 0.767(95% CI, (0.635,0.899); the AUC of GNRI was 0.491 (95% CI, 0.328,0.654); the AUC of SII was 0.544 (95% CI, 0.378,0.709); the AUC of PNI was 0.451 (95% CI, 0.287,0.615). NC, neoadjuvant chemotherapy; NIC, neoadjuvant immunochemotherapy; PNI, prognostic nutritional index; GNRI, geriatric nutritional index; SII, systemic immune-inflammatory index; ROC, Receiver operating characteristic curve; AUC, area under the curve; CI, Confidence interval.

The area under the curve (AUC) of the receiver operating characteristic (ROC) curve for the NC model was 0.794 (95% CI, 0.712, 0.876) in the training set, which was significantly greater than the performances of the GNRI, SII and PNI. Similarly, in the NIC model, the AUC of the ROC curve was 0.781 (95% CI, 0.705, 0.858), which was also better than those of the other three nutritional indicators (**Table 6**, **Figure 3A**). In the validation set, the AUC of the prediction model reached 0.788 (95% CI, 0.664, 0.913) for NC and 0.767 (95% CI, 0.635, 0.899) for NIC. Furthermore, the NC and NIC models performed better than did the GNRI, SII and PNI models in the validation cohort (**Table 6**, **Figure 3C**).

**Table 6:** Comparison of performance with GNRI, SII and PNI.

Variables	Train set		Validation set	
	AUC	(95%CI)	AUC	(95%CI)
Neoadjuvant chemotherapy				
Prediction model	0.794	(0.712,0.876)	0.788	(0.664,0.913)
GNRI	0.539	(0.411,0.638)	0.573	(0.414,0.733)
SII	0.553	(0.445,0.661)	0.438	(0.271,0.606)
PNI	0.645	(0.541,0.749)	0.654	(0.502,0.806)
Neoadjuvant immunochemotherapy				
Prediction model	0.781	(0.705,0.858)	0.767	(0.635,0.899)
GNRI	0.502	(0.409,0.595)	0.491	(0.328,0.654)
SII	0.492	(0.397,0.588)	0.544	(0.378,0.709)
PNI	0.574	(0.479,0.670)	0.451	(0.287,0.615)

PNI, prognostic nutritional index; GNRI, geriatric nutritional index; SII, systemic immune-inflammatory index; AUC, Area under curve; CI, Confidence interval.

**Figure 4:**
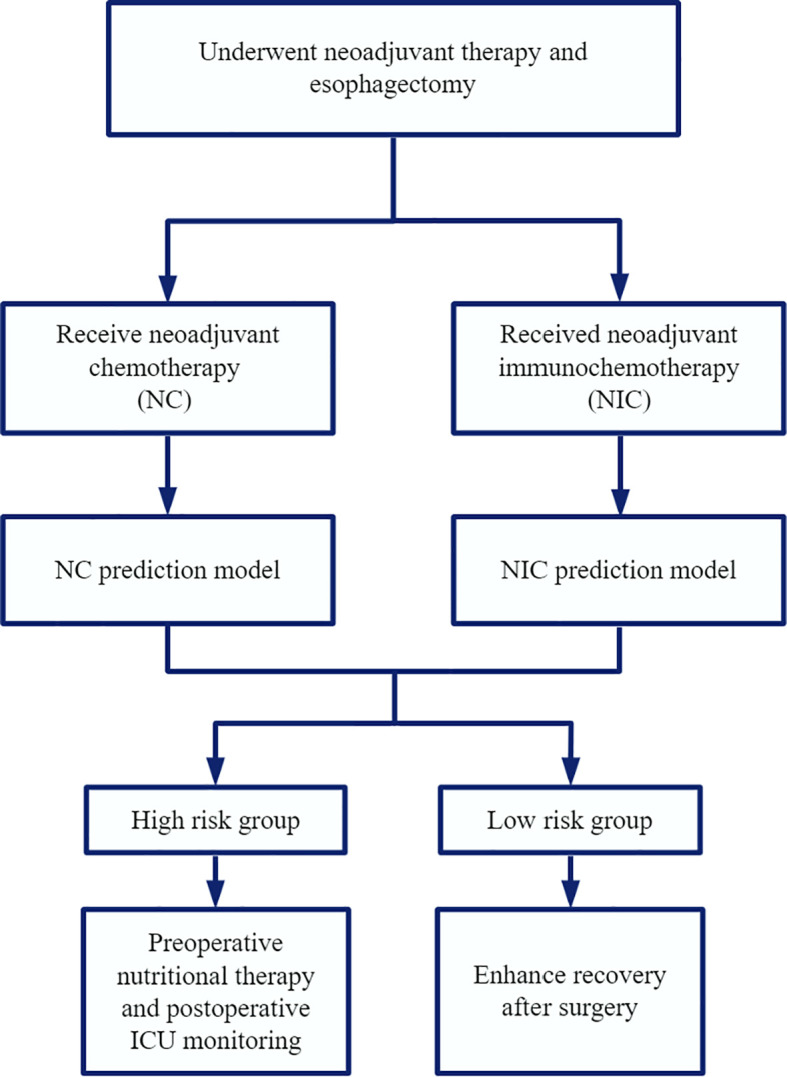
Proposal management for calculated scores deriving from patient information. NC, neoadjuvant chemotherapy; NIC, neoadjuvant immunochemotherapy; ICU, Intensive care unit.

**Figure 5:**
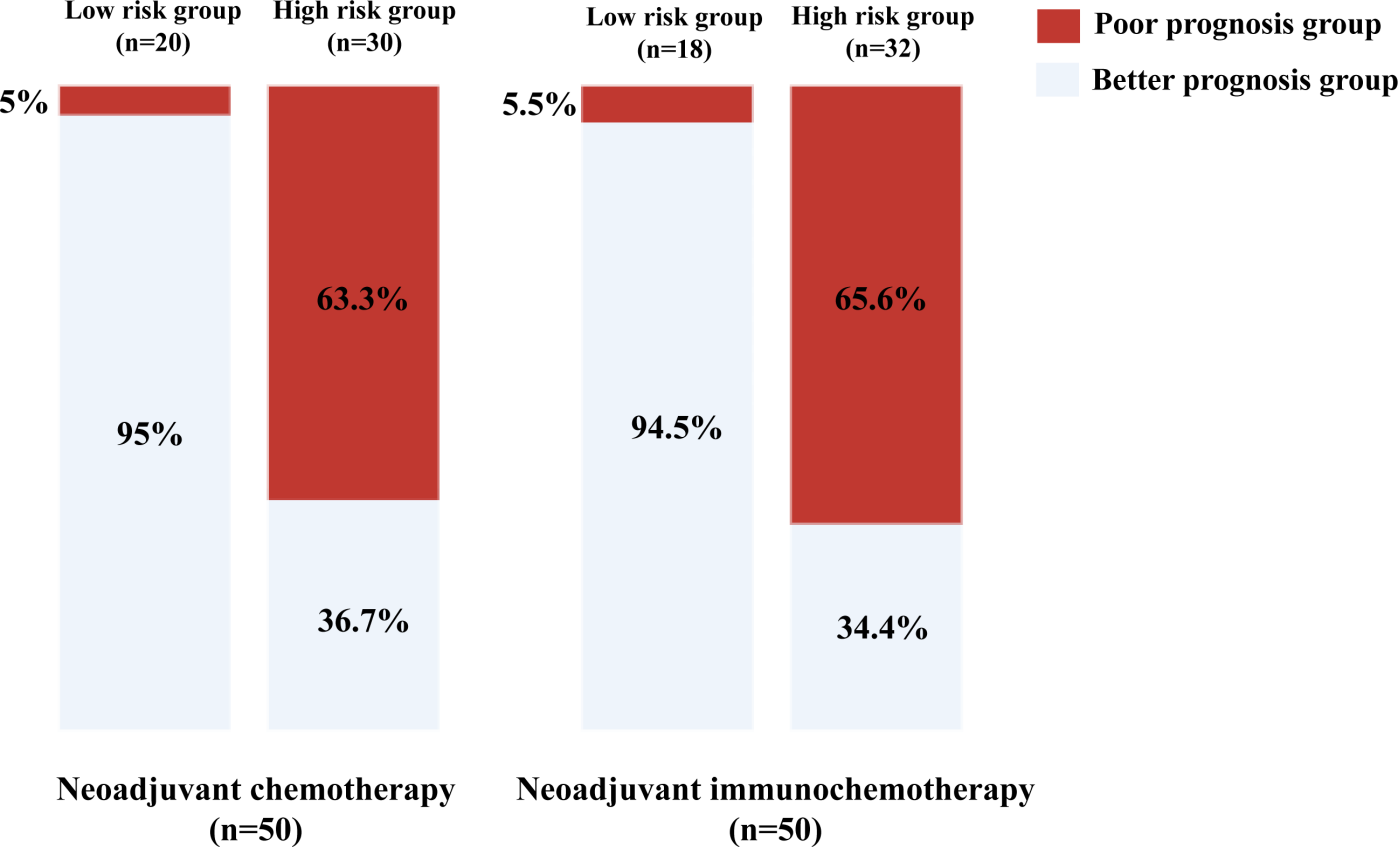
Validation of prediction model in different status.

We further divided the patients into low-risk groups and high-risk groups based on the medium (-10.1927 for NC, -0.4246 for NIC) risk score: the low-risk group (risk scores ≤ medium) and the high-risk group (risk score > medium). To better guide clinical application, we constructed a flow chart for the use of predictive models (**Figure 4**). In the validation set of the NC group, only 1 patient (5%) in the low-risk group was in the poor prognosis group. However, in the high-risk group, the percentage of patients with poor prognosis dramatically increased to 63.3% compared with that in the other groups. Similarly, for NIC patients, there was only 1 low-risk patient in the poor prognosis group, and 65.6% of the high-risk patients were in the poor prognosis group (**Figure 5**).

## Discussion

Postoperative management of esophagectomy after neoadjuvant therapy has always been a major challenge in clinical practice. With the development of neoadjuvant immunochemotherapy (5, 18), the postoperative management of different neoadjuvant treatment methods has also brought new challenges. Various postoperative complications, such as anastomotic leakage, pneumonia, and liver and kidney insufficiency, greatly delay patient recovery and cause perioperative mortality to reach 1.5-3.4% (19, 20). It may also be associated with decreased long-term survival after esophagectomy (21). Therefore, with the development of postoperative management and rapid recovery surgery, distinguishing between patients with poor and better prognoses has created challenges for clinicians.

Given that the poor short-term prognosis of esophagectomy is associated with multiple postoperative complications, most patients develop more than one postoperative complication (21). It is difficult to objectively evaluate the short-term prognosis of patients by analyzing and predicting a certain postoperative complication alone. On this basis, our study incorporated many complications through PCA, an unsupervised dimensionality reduction method, to evaluate the objective progress of postoperative rehabilitation (8, 9). After PC score calculation and further grouping, the short-term prognosis of patients in the better prognosis group and poor prognosis group could be accurately evaluated.

Multivariate analysis revealed that among patients who received neoadjuvant chemotherapy before esophagectomy, only the serum ALB concentration and operation time were found to be independent risk factors for poor prognosis. During the progression of esophageal cancer, the patient’s nutritional status changes with the progression of tumor cells. Poor nutritional status will reduce the patient’s tissue repair ability and immune level, which will lead to poor postoperative recovery (7, 22, 23). Albumin levels can reflect the nutritional status of esophageal cancer patients and are therefore associated with severe postoperative complications and postoperative recovery (24–27). Moreover, a longer surgical time indicates increased surgical difficulty, which is also closely related to higher postoperative complication rates (28). Therefore, the operative time for the poor prognosis group was significantly longer for patients who received neoadjuvant chemotherapy. The area under the curve (AUC) of this neoadjuvant chemotherapy prediction model was 0.794 (95% CI: 0.712, 0.876) in the training set and 0.788 (95% CI: 0.664, 0.913) in the validation set, which were much greater than the other nutritional indices reported in previous studies. It has more advantages in the comprehensive judgment of short-term prognosis.

For patients receiving neoadjuvant immunochemotherapy, urban registration, ASA score, lymphocyte count, number of lymph nodes dissected, lower cholesterol level and higher triglyceride level were found to be independent risk factors for poor prognosis and were completely different from those in the neoadjuvant chemotherapy group. There are differences in the pharmacological mechanisms of neoadjuvant immune combination chemotherapy and neoadjuvant chemotherapy, which may lead to different risk factors (29). Previous studies have shown that urban registration is a risk factor for poor prognosis (30). Similarly, in our study, patients with urban registration had worse short-term prognoses. This may be because the diet of urban patients is less healthy, which affects postoperative recovery. This is also reflected in high triglyceride levels, whose association with postoperative complications such as chylothorax has been confirmed by previous studies (31). In contrast, high cholesterol was a protective factor for patients in the poor prognosis group. This may be because the cell membrane is composed of cholesterol, which plays a role in maintaining cell structure and function. A reduction in cholesterol may impair cell repair function and lead to postoperative complications (32). Moreover, lymphocytes are closely related to immune responses. Low lymphocyte counts have been associated with postoperative complications in patients receiving immunotherapy, both in previous studies and in our study (33, 34). Furthermore, during surgery, a lower ASA score, a clearer anatomical level and a better physical foundation can not only reduce postoperative complications but also increase the number of lymph nodes dissected. This may result in greater lymph node dissection and lower ASA scores being protective factors against postoperative complications. The area under the curve (AUC) of the prediction model for neoadjuvant immunochemotherapy was 0.781 (95% CI: 0.705, 0.858) in the training set and 0.767 (95% CI: 0.635, 0.899) in the validation set, which also indicated good prediction performance for short-term prognosis.

With respect to the prediction model we constructed, compared with the prediction models for short-term prognosis in previous studies, our prediction model targets patients who received neoadjuvant therapy before surgery and provides different patient models for different neoadjuvant treatment approaches (35, 36). While comprehensively predicting and judging short-term prognosis, it also manages patients more accurately. In addition, compared with existing nutritional indicators (GNRI, PNI and SII), while indicators such as GNRI, PNI and SII have been shown to be of value in the prediction of post-esophagectomy complications, our model also achieved better results in predicting short-term prognosis, with higher AUCs and better predictive breadth, which improves the management of patients who undergo esophagectomy.

This study has several limitations. First, this was a retrospective study conducted at three centers. In addition, the model’s training set sample size is relatively small, and the validation set samples are still collected continuously in two centers. In the future, we will design further prospective multicenter studies to confirm these findings, establish causal relationships, and further explore the mechanisms underlying these risk factors.

## Conclusion

A novel short-term outcome prediction model that incorporates diverse perioperative parameters and predicts multiple postoperative complications offers a comprehensive assessment of posttreatment recovery in esophagectomy patients after neoadjuvant immunochemotherapy and neoadjuvant chemotherapy and will be helpful for guiding postoperative management and fast tract recovery.

## Data Availability

The raw data supporting the conclusions of this article will be made available by the authors, without undue reservation.
