# Supplementary figures and images for: A novel short-term outcome prediction model for esophagectomy patients receiving neoadjuvant immunochemotherapy and neoadjuvant chemotherapy: a muti-center retrospective study
Source: Front Oncol. 2026 May 29;16:1805033. doi: 10.3389/fonc.2026.1805033 (PMC13259800; doi:10.3389/fonc.2026.1805033)

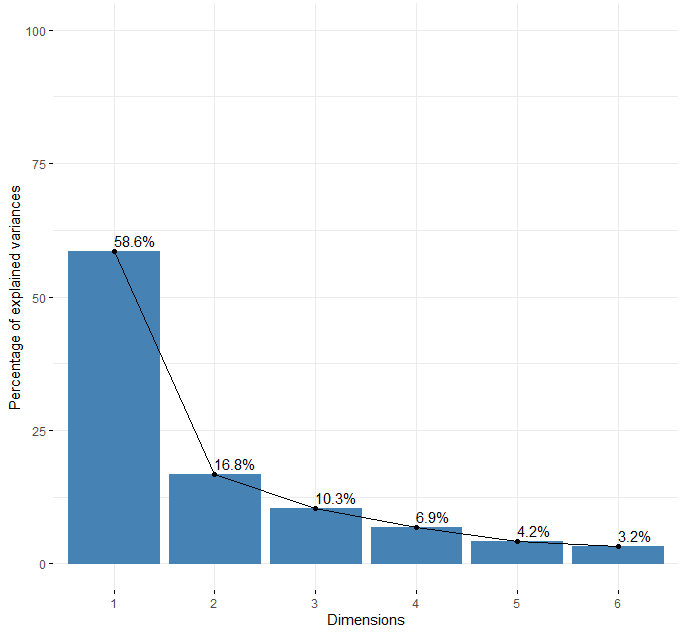

Supplement: Supplementary file 1 [file Image1.png]
